# Characterising and Addressing the Psychosocial Impact of Tuberculosis in Indonesia (CAPITA): A study protocol

**DOI:** 10.12688/wellcomeopenres.17645.2

**Published:** 2022-12-07

**Authors:** Ahmad Fuady, Agus Fitriangga, Agus Sugiharto, Bustanul Arifin, Ferdiana Yunita, Finny Fitry Yani, Helmi Suryani Nasution, I Wayan Gede Artawan Eka Putra, Saidah Rauf, Muchtaruddin Mansyur, Tom Wingfield

**Affiliations:** 1 Department of Community Medicine, Faculty of Medicine, Universitas Indonesia, Pegangsaan Timur No 16 Jakarta, 10310, Indonesia; 2 Department of Community Medicine, Faculty of Medicine, Universitas Tanjungpura, Pontianak, 78124, Indonesia; 3 Faculty of Pharmacy, Universitas Hasanuddin, Makassar, 90245, Indonesia; 4 Department of Community Medicine, Faculty of Medicine, Universitas Gunadarma, Depok, 16451, Indonesia; 5 Faculty of Medicine, Department of Child Health, M. Djamil Hospital, Universitas Andalas, Padang, 25129, Indonesia; 6 Faculty of Medicine and Health Sciences, Universitas Jambi, Jambi, 36122, Indonesia; 7 Department of Public Health and Prevention Medicine, Faculty of Medicine, Universitas Udayana, Bali, 80361, Indonesia; 8 Politeknik Kesehatan Kemenkes Maluku, Maluku, 97711, Indonesia; 9 South East Asian Ministers of Education Organization Regional Center for Food and Nutrition, Jakarta, 13120, Indonesia; 10 Department of Clinical Sciences and International Public Health, Liverpool School of Tropical Medicine, Liverpool, L3 5QA, UK; 11 Department of Global Public Health, WHO Collaborating Centre on Tuberculosis and Social Medicine, Karolinska Institute, Stockholm, 171 76, Sweden; 12 Tropical and Infectious Disease Unit, Royal Liverpool and Broadgreen University Hospitals NHS Trust, Liverpool, L7 8XP, UK

**Keywords:** stigma, discrimination, mental illness, tuberculosis, quality of life

## Abstract

**Background:** Tuberculosis (TB)-related stigma remains a key barrier for people with TB to access and engage with TB services and can contribute to the development of mental illnesses. This study aims to characterise stigmatisation towards people with TB and its psychosocial impact in Indonesia.

**Methods:** This study will apply a sequential mixed method in two main settings: TB services-based population (setting 1) and workplace-based population (setting 2). In setting 1, we will interview 770 adults with TB who undergo sensitive-drug TB treatment in seven provinces of Indonesia. The interview will use the validated TB Stigma Scale questionnaire, Patient Health Questionnaire-9, and EQ-5D-5L to assess stigma, mental illness, and quality of life. In Setting 2, we will deploy an online questionnaire to 640 adult employees in 12 public and private companies. The quantitative data will be followed by in-depth interview to TB-related stakeholders.

**Results:** CAPITA will not only characterise the enacted stigma which are directly experienced by people with TB, but also self-stigma felt by people with TB, secondary stigma faced by their family members, and structural stigma related to the law and policy. The qualitative analyses will strengthen the quantitative findings to formulate the potential policy direction for zero TB stigma in health service facilities and workplaces. Involving all stakeholders, i.e., people with TB, healthcare workers, National Tuberculosis Program officers, The Ministry of Health Workforce, company managers, and employees, will enhance the policy formulation. The validated tool to measure TB-related stigma will also be promoted for scaling up to be implemented at the national level.

**Conclusions:** To improve patient-centered TB control strategy policy, it is essential to characterise and address TB-related stigma and mental illness and explore the needs for psychosocial support for an effective intervention to mitigate the psychosocial impact of TB.

## Introduction

The global community continues to face a high burden of tuberculosis (TB) with a current slow decline of TB incidence and mortality ^1^. This scenario has been aggravated by the coronavirus disease 2019 (COVID-19) pandemic, which continues to negatively impact on the provision of essential TB services ^2, 3^. Amidst these challenging conditions, a key barrier to accessing and engaging with TB services, which must be overcome to end TB globally, remains: stigma ^4, 5^.

TB is a highly stigmatised and stigmatising disease. This is due to visible features associated with TB disease including weight loss, a misconception that TB is incurable, and its association with other stigmatising conditions such as poverty and human immunodeficiency virus (HIV). TB-related stigma contributes to undetected TB cases, delays in accessing timely TB diagnosis and care, poor TB treatment adherence, reduced likelihood of TB treatment success ^6, 7^, and development of drug-resistant TB ^8– 10^. Moreover, TB-related stigma may negatively impact employment opportunities for those affected. People with TB may not be hired because of their illness or a history of TB. They may also receive discrimination in the workplace such as unfair dismissal (sometimes because of unavoidable periods of absence), limited opportunity to advance training and promotion, and avoidance by management and co-workers ^11, 12^. Such stigmatisation and discrimination in workplaces can also compound the psychosocial impact of TB including fear of, or realisation of, social isolation and/or rejection. In turn, these factors can contribute to the development of mental illnesses, such as depression and anxiety ^13^, and their quality of life ^14^.

Indonesia is a high TB burden country with 562,049 new TB cases in 2019. Despite this, there is limited evidence concerning risk factors and prevalence of the psychosocial consequences of TB including stigma, discrimination, depression, and other mental illness ^15^, as well as the interventions to mitigate them. To date, there have been only a few reports from Indonesia on the local and national scenario concerning TB-related stigma ^9, 16, 17^. There is a pressing need for timely interventions to reduce stigma and combat mental illness amongst people with TB to enable them to access available services and achieve better health, psychosocial, and economic outcomes. It is also critical to identify stigmatisation and discrimination towards people with TB in the workplace. This data will be essential to design, develop, and implement locally-appropriate, acceptable, and impactful integrated psychosocial interventions for TB-affected households that address mental illness and stigma and to develop zero stigma and discrimination policies for the workplace ^18, 19^. The *Characterising and Addressing the Psychosocial Impact of Tuberculosis in Indonesia* (CAPITA) study will fill this knowledge gap and provide scientific evidence for further policies to mitigate the psychosocial impact of TB.

## Aims

CAPITA aims to assess (1) stigmatisation and mental health problems experienced by people with TB, (2) assess stigmatisation and discrimination towards people with TB in the workplace, and (3) explore the current national policy on TB-related stigma and identify potential future refinements of this policy.

## Study design overview

CAPITA is a sequential mixed method study consisting of a quantitative approach followed by a qualitative approach using structured in-depth interviews, from February 2022 to February 2023. There will be two main settings in this study. The first setting is TB services-based population to answer aims (1) and (3). The second setting is workplace-based population to answer aims (2) and (3).

## Setting 1: TB services-based population

### Inclusion and exclusion criteria

In this setting, we will include adult (aged 18 years old and above) people with TB who are currently taking drug-sensitive (DS) TB treatment (either first or repeated first-line TB treatment) at public and private primary healthcare facilities that have provided free TB services under coordination of the Indonesian National Tuberculosis Program (NTP). There is substantial evidence on the psychological impact on people with drug-resistant (DR) TB ^15, 20– 22^ but limited evidence among people with DS-TB ^23^. This study will focus on measuring the psychosocial impact on people receiving DS-TB treatment in the primary care setting. People with DR-TB are referred to specific DR-TB treatment facilities (secondary-level hospitals or designated primary care facilities with suitable skills and training). Therefore, the number of people with DR-TB being treated in the primary care setting and/or hospitals without DR-TB treatment provision, would be very low. For this reason, we will exclude people with DR-TB. We will also exclude those who have completed TB treatment, whose responses could be influenced by recall bias.

### Sampling procedure

For this setting, CAPITA will be conducted in seven provinces which represent western (West Sumatera, Jambi, and Jakarta provinces), central (Bali, West Kalimantan, and South Sulawesi provinces), and eastern part of Indonesia (Maluku province). In each province, we will first pragmatically and purposively select two districts that are listed among those with the top five highest TB incidence in the province. Similar to HIV-related stigma, there may be differential TB-related stigmatisation across rural and urban areas. For example, perceived or anticipated TB-related stigma may be greatest amongst people who live in urban areas ^24, 25^, but psychological distress because of TB may more likely occur among those living in rural areas ^26, 27^. To obtain a clearer picture, we will select two sub-districts representing urban areas in one district while, in another district, we will select two sub-districts representing rural areas according to the Indonesian National Statistical Bureau ( *Badan Pusat Statistik,* BPS) classification ^28– 30^. Jakarta, a capital of Indonesia, is an exception since all subdistricts in the province are classified as urban areas. In this case, we will select the four sub-districts according to the BPS estimates of their population density (very high and high density) ^30^. The selection of all sub-districts will be consulted with the NTP program officers in province and district level.

The NTP program officers in district level will appoint a contact person in sub-district level who will help check the patient database. We will then interview the subjects who are selected according to inclusion and exclusion criteria, either face-to-face, phone, or online interview. The responses will be stored electronically and monitored through REDCap®, a secure web application for managing online surveys ^31^.

### Sample size determination

This study will follow the STOP-TB Partnership’s recently published TB Stigma Assessment Implementation Handbook ^13^ and use a locally-adapted version of the TB-related stigma questionnaire by Van Rie (2008) ^32^. Based on Van Rie’s study in Thailand, the average stigma score of patient perspectives toward tuberculosis was 27.6 with standard deviation of 6.1 ^32^. Using an alpha of 0.05, acceptable absolute precision 0.7, and a doubled-power because of urban-rural stratification, we need at least 584 subjects in this study for generalization.

We will consult with the National TB Program Registers at each health facility and select respondents consecutively, starting from those most recently diagnosed. We will divide the respondents based on their TB treatment status. First, people who are in the initial two months of a standard six-month first DS-TB regimen (intensive treatment phase). Second, people who are receiving TB treatment in private healthcare facilities. Third, people who were diagnosed with TB at public facilities but did not start TB treatment, termed “lost to follow up to treatment”. We assume that people with TB across these three groups, but especially in the second and third groups, may experience TB-related stigma. Since the number of people in the second and third groups are lower than the number of those in first group, we will apply a recruitment ratio of 3:2 (first group:second+third group).

In addition to these three groups, we also assume that, amongst other factors, stigmatisation and mental health problems may lead people with TB to discontinue the treatment or be lost to follow up (LTFU), which have recently been measured as five to 16% ^17, 33^. These people who have discontinued or been LTFU are then potentially re-treated at a later date. For this reason, we will select five patients receiving a six-to-nine-month DS-TB re-treatment regimen, at any phase of their treatment (see Table 1). In total, we will include 770 people with TB in this study. If the completed responses are still less than 98% of the expected sample size in one subdistrict after one month, and there are no other people with TB registered in that subdistrict, we will expand the sampling area to another subdistrict which has similar characteristics.

**Table 1.** Sample allocation in each province.

Province	Urban sub-district			Rural sub-district			Total
	Receiving TB treatment in the intensive phase	Lost to follow up to treatment or receiving treatment at private facilities	Receiving TB re-treatment	Receiving TB treatment in the intensive phase	Lost to follow up to treatment or receiving treatment at private facilities	Receiving TB re-treatment	
Jambi	30	20	5	30	20	5	110
West Sumatera	30	20	5	30	20	5	110
Jakarta	30	20	5	30	20	5	110
West Kalimantan	30	20	5	30	20	5	110
Bali	30	20	5	30	20	5	110
South Sulawesi	30	20	5	30	20	5	110
Maluku	30	20	5	30	20	5	110
**Total**	**210**	**140**	**35**	**210**	**140**	**35**	**770**

### Instruments

We will first do a cross-cultural adaptation of the TB Stigma Scale questionnaire ^32^ and validate it to the Indonesian context. To measure mental health impact among TB-affected people, this study will use the Patient Health Questionnaire-9 (PHQ-9), which has previously been applied and validated in Bahasa Indonesia ^34, 35^. This study will also inform the design of future psychosocial intervention. Therefore, we add ten questions to measure respondents’ needs for psychosocial support, such as home visit, community meeting, and counseling (see Supplement A in the *Extended data* ^36^).

The cultural adaptation of the TB Stigma Scale questionnaire will follow the The Professional Society for Health Economics and Outcomes Research (ISPOR) Framework: forward translation, reconciliation, back translation, harmonization, cognitive debriefing (piloting), proofreading, and finalisation ^37^. All combined questionnaires will be harmonized in an expert panel meeting involving experts from community medicine, psychiatry, pulmonology, the Indonesian NTP, people with TB, and civil society representatives. The final questionnaire will be field-tested on 20 participants prior to study initiation. The field-testing will include cognitive debriefing to test wording used in the questionnaire and to check understandability, interpretation, and cultural relevance of the translation. The results will be finalized before implementation.

At the end of interview, people with TB will be also asked set of questions of the EQ-5D-5L questionnaire to measure their health-related quality of life ^38^. This questionnaire consists of five dimensions: mobility, self-care, usual activities, pain/discomfort, anxiety/depression with five levels of severity. We will also add the EQ-5D-3L questionnaire to compare the sensitivity, reliability, and ceiling effects between three (3L) and five levels (5L) of severity. Both questionnaires have been applied and validated in the Indonesian context ^39^.

### Desk review

We will do a desk review to explore the current national policy on TB-related stigma. The review will include Indonesian Law (Undang-Undang), President Regulation (Peraturan Presiden), President Decree (Keputusan Presiden), Ministry Regulations (Peraturan Menteri), Ministry Decrees (Keputusan Menteri), and technical regulation within ministries, issued between 2000-2022. The review will be summarized in a table and the synthesis results will be a basis for probing questions in in-depth interview.

### In-depth interview

To complement the findings from questionnaires with people with TB, we will apply a qualitative approach to explore the current policy concerning stigma towards people with TB. The approach will be applied through in-depth interviews with purposively selected key stakeholders: six NTP program officers in sub-district level, six TB program officers/healthcare workers in Puskesmas level, 12 people with TB, and 12 household contacts of people with TB. This sample size of 36 participants has been selected as it is perceived to provide sufficient information power. Those who are invited to the in-depth interview will be selected purposively according to sub-district area (urban/rural), and we will ensure gender and age balance where possible. The in-depth interview will be held via online discussion, using a structured open-ended questionnaire.

## Setting 2: Workplace-based population

To assess TB-related stigma in workplaces and explore the current TB-related stigma policy in workplaces and its further direction, CAPITA Study will target employees who work in either formal or informal sector as the second study population.

### Inclusion and exclusion criteria

In this setting, we will include adults (aged 18 years and above) who work at any level of employment in either a public or private enterprises in the either formal or informal sector and agree to participate in the study. There are no specific exclusion criteria for this population.

### Sampling procedure

To recruit the respondents, we will first select enterprises purposively from a network database in the Master Program of Occupational Medicine, Faculty of Medicine, Universitas Indonesia and its alumni network, as well as Indonesian TB research network. In this study, enterprises in the formal sector are defined as “medium to large size enterprises” with more than 50 employees and formally registered with the Ministry of Investment/Indonesian Investment Coordinating Board. These include manufacturing, wholesale, and service enterprises. Enterprises in the informal sector are defined as “small size enterprises with less than 50 employees and not formally registered with the Ministry of Investment/Indonesian Investment Coordinating Board. These small enterprises include the home industries such as bag makers, bakers, home chip makers, and local farmers. At each enterprise, we contacted the human resource department (formal sector) and the owner (informal sector) to select division(s) or group(s) of workers aged >18 years old.

The enterprises will be selected based on the number of employees, developed by the National Statistical Bureau: 100-250 employees and 250-500 employees ^40^. We will contact the department of human resources at each enterprise to help share either online or paper-based questionnaire to their employees—either at manager or staff level. The responses will be monitored daily from the REDCap®.

### Sample size determination

In this setting, we use the same assumptions applied for setting 1. According to Van Rie’s study in Thailand, the average stigma score of community perspectives toward tuberculosis was 27.9 with standard deviation of 6.1 ^32^. We apply a 5% of error and acceptable absolute precision 0.7, but without double power since we will not apply stratification to this population. Therefore, this study needs at least 292 subjects for generalizability ( Table 2).

**Table 2.** Sample allocation for workplace-based population.

Enterprise size	# of enterprises	# of participants per enterprise	Total
Informal sector enterprises	Minimum 2		50
Formal sector enterprises			
50-250 employees	3	45	135
250-500 employees	3	70	210
**Total**	**11**		**560**

### Instruments

In this setting, we will develop a self-administered questionnaire, based on TB Stigma Scale and TB Stigma Assessment Handbook (see Supplement B in the *Extended data* ^36^.) ^14, 32, 42^ We will validate the questionnaire’s content through an expert panel meeting consisting of experts from community medicine, psychiatry, occupational medicine or health management, the Indonesian NTP, and Ministry of Health (Directorate of Occupational Health). The content-validated questionnaire will be field-tested and finalized as also implemented for setting 1.

### In-depth interview

We will also apply a qualitative approach through in-depth interviews. Using probing questions developed from a desk review, the interviews with selected key persons will: two director-level resource persons at the Ministry of Workforce, two director-level resource persons at the Ministry of Health (Occupational Health and Infection), six manager-level at six different enterprises, and 12 non-manager level workers from six enterprises (two workers per enterprise). Those who are invited to the in-depth interview will be selected purposively according to their availability. The in-depth interview will be held via online discussion, using a structured open-ended questionnaire, and will last around 45 minutes. The questions will be finalized after obtaining quantitative results and an expert panel meeting.

## Data management

Data management of the study will be performed under the responsibility of investigators. Investigators will develop an electronic-based questionnaire using REDCap® for the collection of field data and real-time monitoring of the implementation of the online surveys. For qualitative data, all in-depth interviews will be recorded electronically and stored in cloud platform with a unique password. The records are also stored in local drive for backup. The sound records will be transcribed, and all will be stored at cloud platform with unique password.

The collected data will be anonymized. There will be no name and address of the subjects. Investigators are the only persons who have access to individual anonymized data. Access to the physical and electronic data is only granted by the Principal Investigator (PI). All passwords are strong in nature and never written down nor shared.

The study database will be locked as soon as it is considered clean. Only authorized and well-documented updates to the study data are possible after database lock. The locked database is used in the final statistical analysis for study reporting. Measures will be undertaken to protect subject data handed over by the Investigator to the data management department and during inspections against disclosure to unauthorized third parties. Subject confidentiality will always be maintained.

## Outcome analyses

The outcome in this study is psychosocial impact, defined by measurement of stigma, mental health, and health-related quality of life. We will also measure the respondents’ needs for social support.

### Stigma

Stigma will be scored numerically by a four number Likert scale from strongly disagree (1) to strongly agree (4). The mean sum of scores across the questions and accompanying SD will be displayed. For validation, we will apply exploratory and confirmatory factor analysis and internal consistency analysis. A Cronbach’s Alpha of >0.7 will be considered good to excellent reliability or internally consistent. We will also check the construct validity by calculating Pearson’s correlation coefficients to compare individual scores on the Van Rie’s stigma scales with responses concerning mental health (PHQ-9). We will consider a correlation coefficient >0.3 indicated moderate to strong correlation. We hypothesize that TB-related stigma will be positively correlated with social support need and mental health. Significance level will be set at 0.05.

### Mental health

We will calculate the sum value of each subject and display the score in five groups: from minimal to severe depression. However, for further inferential analysis, we will group the scores to only three categories ( Table 3).

**Table 3.** Interpretation of Patient Health Questionnaire-9 (PHQ-9) total score.

Total score	Interpretation 1	Interpretation 2
1–4	Minimal depression	No to minimal depression
5–9	Mild depression	Mild to moderate depression
10–14	Moderate depression	
15–19	Moderately severe depression	Severe depression
20–27	Severe depression	

### Health-related Quality of Life (HRQoL)

Each dimension of the EQ-5D-5L has five-level options; from 1 for ‘no problem’ to 5 for ‘unable or extremely problem’ while the EQ-5D-3L has only three levels. Therefore, the answer will be coded as five numbers, for example, 11111 or 12345 for EQ-5D-5L and 111 or 123 for EQ-5D-3L. The codes will be converted to utility scores validated in Bahasa Indonesia ^36^. Both questionnaires will be compared to see the reliability and ceiling effects with the EQ-5D-3L. Quality of life data will be displayed in mean (standard deviation, SD) and/or median (min-max) values. Ceiling will be displayed in number (n) and percentages (%).

### Need for social support

In this study, we will develop an instrument to measure whether respondents perceive that they have received information support, emotional support, and/or instrumental support ^40^. ( Table 4) Information support consists of education (including information about the etiology, prevention and treatment of the disease) provided by healthcare staff for either the people with TB or their family members including through one-to-one individual or peer group consultations. Emotional support includes empathy, being listened to, and being given the opportunity to talk with healthcare staff, family members, and other people with TB. Instrumental support includes home visits, peer group meetings, and personal and group counseling.

**Table 4.** Instrument to measure social support received and needed by people with TB.

Social support	I perceive that I…				Needs	
	Had never received	have not received enough	Have received enough	Have received a lot	Yes, I need	No, I don’t need
**Information support**						
Education about TB from healthcare staff, for me	[ ]	[ ]	[ ]	[ ]	[ ]	[ ]
Education about TB from healthcare staff, for my family members	[ ]	[ ]	[ ]	[ ]	[ ]	[ ]
Education about TB from healthcare staff, for me, in peer-group meeting	[ ]	[ ]	[ ]	[ ]	[ ]	[ ]
**Emotional support**						
Emotional support from healthcare staff	[ ]	[ ]	[ ]	[ ]	[ ]	[ ]
Emotional support from family members	[ ]	[ ]	[ ]	[ ]	[ ]	[ ]
Emotional support from peer with TB	[ ]	[ ]	[ ]	[ ]	[ ]	[ ]
**Instrumental support**						
Home visit by healthcare staff	[ ]	[ ]	[ ]	[ ]	[ ]	[ ]
Peer-group meeting with people with TB	[ ]	[ ]	[ ]	[ ]	[ ]	[ ]
Individual psychological counseling, for me	[ ]	[ ]	[ ]	[ ]	[ ]	[ ]
Group counseling with other people with TB	[ ]	[ ]	[ ]	[ ]	[ ]	[ ]

### Association between variables

We will assess the correlation between TB-related stigma, need for social support and HRQoL by calculating Pearson’s or Spearman-rank correlation, depending on their data distribution. Factors related to TB-related stigma, mental health, needs for support, and HRQoL will also be assessed. The presumptive demographic factors include age, sex, education level, economic/poverty level, and area of residence, while clinical factors include type of TB (e.g. first vs recurrent drug sensitive-TB), HIV co-infection, and phase of TB treatment. All these factors will be included as fixed factors in ANOVA (univariate analysis) and MANOVA (in multivariate analysis) to obtain the F, partial η ^2^ and significance values. For variables with more than two categories, we will perform complementary post-hoc analyses using Tukey’s honest significant test (HSD) when the data meet the Tukey’s assumption, or Bonferroni correction as a suitable alternative. Significance will be set at 5%. All statistical analyses will be done using SPSS 27.0.

### Qualitative analysis

All in-depth interviews will be recorded after interviewees give their consent. The recordings will be then transcribed to text. Keywords found in the transcript will be coded and grouped according to the theme, using Microsoft Excel. A content analysis will be done using three steps summarized below ^44^.

a. *Summary*. Given the long transcripts, we will do paraphrasing, generalisation, or abstraction to summarize the text to preserve the essential content but still reflects the original material.
b. *Explication*. The material then will be explained, clarified, and annotated by (1) defining it lexico-grammatically and (b) determining the material for explication, then followed by (c) a narrow and broad context analysis.
c. *Structuring*. We will first determine the themes for thematic analysis. This step will be done independently using multiple coders by two researchers who familiarised themselves with the data. The themes and codes will be re-examined and revised, if necessary, depending on the course of reappraisal of the material.

We will do triangulation in content analysis by comparing findings from several perspectives: patients – healthcare providers – program officers (for TB service-based population) and employees – enterprise management – policy makers (for workplace-based population), from either qualitative or quantitative findings. The findings will be displayed in several themes, and some quotes will be provided in the narration.

## Ethical considerations

CAPITA study’s protocol will be submitted to The Ethical Research Committee, The Faculty of Medicine, Universitas Indonesia and will be undertaken only after the IEC/IRB has given full written approval of the final protocol, the questionnaire, and any other written information to be provided to the subjects.

All subjects will be provided a full explanation on the nature of the study and written informed consent will be taken. All information should be delivered in Bahasa Indonesia. Because of potential COVID-19 waves, interviews can be done through either face-to-face, by phone, or online interview. Therefore, the informed consent form will be signed according to the way of the interview: written for face-to-face and recorded voice or video for phone and online interview. The respondents will authorize such access and agree to allow study team to recontact them to obtain missing or additional data, if needed. Investigators ensure that all study documents are provided in confidence and are not disclosed to any party not directly involved in the study without the investigator’s written permission.

## Discussion

CAPITA Study will be the first study that captures the TB-related stigma, discrimination, and mental health problems experienced by people with TB and their health-related quality of life in several provinces in Indonesia. To date, there has been limited local evidence generated concerning risk factors and prevalence of the psychosocial consequences of TB, including stigma and mental illness, and the interventions to mitigate them. Most reports were published in the grey literature and not peer-reviewed ^15^.

In addition to characterizing the psychosocial impact faced by people with TB, CAPITA Study will also explore the perception among workers regarding the stigmatisation and discrimination towards people at the workplace. There is no previous study assessing this issue in Indonesia. At the same time, people with TB often miss job opportunities or receive discrimination in the workplace, such as unfair dismissal because of repeated absenteeism, limited opportunity to promotion, avoidance, or even being fired from their job ^11, 12, 45^.

With a sequential mixed method, the quantitative findings will be followed by an assessment through a qualitative approach to assess the current policy regarding TB-related stigma, discrimination, and mental illness and explore the further policy direction in health service facilities and workplaces. The qualitative approach will help enhance and check the quantitative findings by triangulation in content analysis from many perspectives: people with TB, NTP managers, TB program officers, The Ministry of Health Workforce, enterprise managers, and employees.

Involving policy makers in the national Indonesian context since the preparation of the study is essential to influence the development of future policy or policy agenda. At the end of the study, we will invite all related stakeholders to a participatory workshop to discuss the findings and plan the next steps for zero TB stigma policy improvement. The results will be formulated in policy briefs for The Ministry of Health and The Ministry of Workforce, Republic of Indonesia, scientific articles published in peer-reviewed journals, and inputs for the WHO’s handbook of TB Stigma, if applicable. We will also hold a public webinar to disseminate the findings to the public audience. In addition, the validated tool to measure TB-related stigma will be promoted for scaling up to be implemented at the national level and used for future research in Indonesia.

This study will be implemented during the ongoing COVID-19 pandemic. Therefore, respondent recruitment, particularly for setting 1, may be challenged by new severe acute respiratory syndrome coronavirus 2 (SARS-CoV-2) waves, TB and healthcare service disruption, or lower TB case notification rates. To mitigate the negative effects of pandemic to this study, flexibility in doing interview and in-depth interview is critical. Using phone or virtual call, for example via Zoom or Microsoft Teams, is both an opportunity and a challenge ^46^. The data validity and comparability collected by different mediums should be maintained through a well-prepared training for interviewers, strong monitoring, and clean data auditing.

We acknowledge the potential limitations of this study. First, in Setting 1, we will not be able to capture the psychosocial impact faced by people with TB who are not notified by the NTP and not detected, which is approximately 20% of total TB incidence in Indonesia. Second, we acknowledge the wide geographical and cultural contexts of Indonesia. Although the seven provinces in this study were not randomly selected, these provinces represent areas with diverse TB burdens in Indonesia. However, the results should be interpreted carefully because it is known that symptoms of stigma and depression may be underestimated in areas with strong local, traditional beliefs ^47^ and low development ^7^. Third, this study will not capture the psychosocial impact of DR-TB, which is a high-risk group for stigma. In setting 2, the responses to the online questionnaire depend on those who are willing to join the survey. These selection biases will limit the generalizability of the findings.

## Study status

In this peer-review stage, the study protocol had been reviewed and approved by The Ethical Committee of Faculty of Medicine, Universitas Indonesia, including the amandment of the protocol (No. KET-60/UN2.F1/ETIK/PPM.00.02/2022). We accomplished the cross-cultural adaptation of the Scale used in this study to the Indonesian context. The data collection has been almost finished.

## Data Availability

No underlying data are associated with this article. Open Science Framework: Characterising and Addressing the Psychosocial Impact of Tuberculosis in Indonesia (CAPITA). https://doi.org/10.17605/OSF.IO/2UB9J ^36^. This project contains the following extended data: d1. Pedoman Lembar Informed Consent-gab af v.2.pdf Supplements – CAPITA.pdf (questionnaires) Data are available under the terms of the Creative Commons Zero “No rights reserved” data waiver (CC0 1.0 Public domain dedication).
