# Supplementary material for: Characterising and Addressing the Psychosocial Impact of Tuberculosis in Indonesia (CAPITA): A study protocol
Source: Wellcome Open Res. 2022 Dec 7;7:42. Originally published 2022 Feb 4. [Version 2] doi: 10.12688/wellcomeopenres.17645.2 (PMC9975400; doi:10.12688/wellcomeopenres.17645.2)
Supplement: Peer review history

# Peer review: Reviewer response for version 2

**DOI:** 10.21956/wellcomeopenres.20674.r53645

**Published:** 2022-12-14

**Authors:** Linh Nguyen Nhat

**Affiliations:** 1 Global TB Programme, World Health Organization, Geneva, Switzerland

I agree with the changes and approve the revised version of the manuscript.

Is the study design appropriate for the research question?

Yes

Is the rationale for, and objectives of, the study clearly described?

Yes

Are sufficient details of the methods provided to allow replication by others?

Partly

Are the datasets clearly presented in a useable and accessible format?

Not applicable

Reviewer Expertise:

Tuberculosis: epidemiology, screening, treatment, care and support.

I confirm that I have read this submission and believe that I have an appropriate level of expertise to confirm that it is of an acceptable scientific standard.

# Peer review: Reviewer response for version 1

**DOI:** 10.21956/wellcomeopenres.19521.r52997

**Published:** 2022-11-08

**Authors:** Soedarsono Soedarsono

**Affiliations:** 1 Department of Pulmonology and Respiratory Medicine, Faculty of Medicine, Dr Soetomo Hospital, Universitas Airlangga, Surabaya, Indonesia

This study aims to characterise stigmatisation towards people with TB and its psychosocial impact in Indonesia. This study has been well written, but there are some statements that needed to be confirmed by the author:

1. Indonesia is a big country with a very wide area and various characteristics of different peoples and ethnic groups. This study was conducted in seven provinces which represent the western (West Sumatera, Jambi, and Jakarta provinces), central (Bali, West Kalimantan, and South Sulawesi provinces), and eastern parts of Indonesia (Maluku province). Is the sampling representative of the characteristics of Indonesian society as a whole, as several large areas in Indonesia have not been included in this study (e.g., East Java, West Java, Papua, etc.), causing this study to be less representative?
2. This study was conducted in rural and urban areas: the number of samples should be adjusted to the ratio of the percentage of rural and urban areas in Indonesia.
3. This study took 30 samples in the intensive phase and 20 in the continuation phase. What is the basis for this number of samples? Because, if we look at it at a glance when treating TB patients, the incidence of loss to follow-up is more often found in the intensive phase than the continuation phase. In the intensive phase, the incidence of loss to follow-up is often caused by various reasons (stigma, etc). While in the continuation phase, the patient seems to be enjoyed undergoing treatment without being afraid of the stigma, as they do not need to come more often to healthcare facilities (once in 2 weeks or 1 month). Therefore, why does this study not only use subjects in the intensive phase?

Is the study design appropriate for the research question?

No

Is the rationale for, and objectives of, the study clearly described?

Yes

Are sufficient details of the methods provided to allow replication by others?

Yes

Are the datasets clearly presented in a useable and accessible format?

Yes

Reviewer Expertise:

Tuberculosis, Drug-Resistant Tuberculosis, Bacterial Pneumonia, Pulmonary Fungal Infection

I confirm that I have read this submission and believe that I have an appropriate level of expertise to confirm that it is of an acceptable scientific standard.

# Response

**Published:** 2022-11-24

**Authors:** Ahmad Fuady

**Affiliations:** University of Indonesia, Indonesia

Dear Dr. Sudarsono,

 Thank you very much for your valuable comments and suggestions. Please kindly find our responses below.

 1. Indonesia is a big country with a very wide area and various characteristics of different peoples and ethnic groups. This study was conducted in seven provinces which represent the western (West Sumatera, Jambi, and Jakarta provinces), central (Bali, West Kalimantan, and South Sulawesi provinces), and eastern parts of Indonesia (Maluku province). Is the sampling representative of the characteristics of Indonesian society as a whole, as several large areas in Indonesia have not been included in this study (e.g., East Java, West Java, Papua, etc.), causing this study to be less representative?

 Response:

 Thank you for the comment. We also acknowledge the broad geographical and cultural contexts of Indonesia. The seven provinces were selected purposively to represent western, central, and eastern part of Indonesia. Despite not being randomly selected, these provinces represent areas with diverse TB burdens in Indonesia. However, the results should be interpreted carefully. Symptoms of stigma and depression may be underestimated in areas with strong local, traditional beliefs (Manurung, 2018) and low development (Datiko, 2020). We have now added this issue as a limitation in the Discussion section.

 2. This study was conducted in rural and urban areas: the number of samples should be adjusted to the ratio of the percentage of rural and urban areas in Indonesia.

 Response:

 Thank you for your suggestion. In this study, we select at least four districts with high TB incidence: two urban and two rural districts. The districts were selected purposively after consultation with the NTP program officers at the province and district levels. Although we do not adjust the ratio of the rural and urban areas, the purposive selection could represent the middle to high TB burden, which often leads to a high rate of stigmatisation due to TB.

 3. This study took 30 samples in the intensive phase and 20 in the continuation phase. What is the basis for this number of samples? Because, if we look at it at a glance when treating TB patients, the incidence of loss to follow-up is more often found in the intensive phase than the continuation phase. In the intensive phase, the incidence of loss to follow-up is often caused by various reasons (stigma, etc). While in the continuation phase, the patient seems to be enjoyed undergoing treatment without being afraid of the stigma, as they do not need to come more often to healthcare facilities (once in 2 weeks or 1 month). Therefore, why does this study not only use subjects in the intensive phase?

 Response:

 Thank you for this comment. We consulted the sampling method with experts and NTP officers, which resulted in changes being made to the sampling method. (Please see revisions under **Setting 1: TB services-based population > Sample size determination**)

 We hope that the revised manuscript has addressed all comments and suggestions and can be proceed further.

 With kind regards,

 on behalf of all co-authors,

 Ahmad Fuady

# Peer review: Reviewer response for version 1

**DOI:** 10.21956/wellcomeopenres.19521.r52803

**Published:** 2022-10-31

**Authors:** Linh Nguyen Nhat

**Affiliations:** 1 Global TB Programme, World Health Organization, Geneva, Switzerland

The manuscript described a study proposal that aims to assess impact of TB-related stigma, discrimination and mental health problems on quality of life of people with TB and explore potential improvements of the national policy on TB-related stigma. The aims and design of the study have been well described. However, some issues in methods and implementation of the study, as listed below, need further clarifications by the authors. 

 - The authors used "psychosocial impact" in the title. However, I wonder if the term "psychosocial" is widely understood or correctly interpreted by the audience/readers. Later in the manuscript, there are description on impact of "TB-related stigma, discrimination and mental health problems" on "health-related quality of life" that are better interpreted than using of "psychosocial impact".

 - The study intentionally excludes drug-resistant TB in the inclusion criteria and participant's recruitment without sufficient explanation of reasons, although drug-resistant TB patients, especially people with MDR-TB, are well-known of facing more stigmatization and mental health problems. At the same time, the study also recruits TB patients on retreatment while many among these patients likely contract drug-resistant TB. Authors would need to clarify the rationale behind recruiting patients on retreatment and not recruiting patients with drug-resistant TB for the study. 

 - It was unclear what kind of companies that are included in the database of the Master Program of Occupational Medicine, Faculty of Medicine. Better descriptions of what kind of companies targeted and included in the study are needed. Similarly, it is not clear what "formal sector" is referred to for the companies selected.

 - Outcome analysis section referred to "need for social support" and this is scored from strongly disagree to strongly agree. However, there is no standardized definition of social support or description on what kind of support included. A widely used "social support" includes information/education support, psychological support and material support (including several types of support such as cash transfer, food package, transport subsidy etc.). A clear description in the method of assessment and outcome analysis would help come up with better outcomes.

Is the study design appropriate for the research question?

Yes

Is the rationale for, and objectives of, the study clearly described?

Yes

Are sufficient details of the methods provided to allow replication by others?

Partly

Are the datasets clearly presented in a useable and accessible format?

Not applicable

Reviewer Expertise:

Tuberculosis: epidemiology, screening, treatment, care and support.

I confirm that I have read this submission and believe that I have an appropriate level of expertise to confirm that it is of an acceptable scientific standard, however I have significant reservations, as outlined above.

# Response

**Published:** 2022-11-24

**Authors:** Ahmad Fuady

**Affiliations:** University of Indonesia, Indonesia

Dear Dr. Linh Nguyen Nhat,

 Thank you very much for your valuable comments and suggestions. Please kindly find our responses below.

 1. The authors used "psychosocial impact" in the title. However, I wonder if the term "psychosocial" is widely understood or correctly interpreted by the audience/readers. Later in the manuscript, there are description on impact of "TB-related stigma, discrimination and mental health problems" on "health-related quality of life" that are better interpreted than using of "psychosocial impact".

 Response:

 Thank you very much for your comment. In this study, we used the term ‘psychosocial impact’ to refer to stigma, discrimination, symptoms of depression, and reduced quality of life. This psychosocial impact is in line with relevant definitions “describing the intersection and interaction of social, cultural, and environmental influences on the mind and behaviour” (APA Dictionary of Psychology). We have provided additional references to the manuscript and further defined the “impacts” under the heading ‘Outcome analyses’. Therefore, given these updates, we would prefer to keep the title using the term “psychosocial impact”.

 2. The study intentionally excludes drug-resistant TB in the inclusion criteria and participant's recruitment without sufficient explanation of reasons, although drug-resistant TB patients, especially people with MDR-TB, are well-known of facing more stigmatization and mental health problems. At the same time, the study also recruits TB patients on retreatment while many among these patients likely contract drug-resistant TB. Authors would need to clarify the rationale behind recruiting patients on retreatment and not recruiting patients with drug-resistant TB for the study.

 Response:

 Thank you very much for the comment. We also acknowledge the evidence that people with drug-resistant TB (DR-TB) experience psychological impact, references for which we include in the manuscript. However, the evidence of psychological impact among people with DS-TB treatment including people who were diagnosed with TB but not following with TB treatment (in this study referred to as people who have been ‘lost to follow up to treatment’) is still limited, especially in Indonesia. We also assume that, amongst other factors, stigmatisation and mental health problems may lead people with TB to discontinue their treatment or be lost to follow up (LTFU). For these reasons, we will recruit people being both newly treated and retreated for TB in the primary care setting.

 People with DR-TB are referred to specific DR-TB treatment facilities (secondary-level hospitals or designated primary care facilities with suitable skills and training). Therefore, the number of people with DR-TB being treated in the primary care setting and/or hospitals without DR-TB treatment provision, would be very low. For this reason, we have excluded people with DR-TB from our study, the enrolment for which is ongoing, and now acknowledge this issue in the limitations section of the protocol.

 3. It was unclear what kind of companies that are included in the database of the Master Program of Occupational Medicine, Faculty of Medicine. Better descriptions of what kind of companies targeted and included in the study are needed. Similarly, it is not clear what "formal sector" is referred to for the companies selected.

 Response:

 We have now added an explanation about the enterprises selected in this study. We have extended the target enterprises to those in both the formal and informal sectors. See the revision under “Setting 2: Workplace-based population”.

 4. Outcome analysis section referred to "need for social support" and this is scored from strongly disagree to strongly agree. However, there is no standardized definition of social support or description on what kind of support included. A widely used "social support" includes information/education support, psychological support and material support (including several types of support such as cash transfer, food package, transport subsidy etc.). A clear description in the method of assessment and outcome analysis would help come up with better outcomes.

 Response:

 After consultation within the interdisciplinary study team and with experts during expert panel meetings, we revised the approach to measuring the need for social support. Social support consists of information, emotional, and instrumental support. We added this explanation in the “Need for social support” section.

 We hope that the revised manuscript has addressed all comments and suggestions and can be proceed further.

 With kind regards,

 on behalf of all co-authors,

 Ahmad Fuady
